# The efficacy of low-dose anlotinib as a first-line treatment for frail patients with lung adenocarcinoma and its bridging significance for subsequent treatment: case report

**DOI:** 10.3389/fmed.2025.1659090

**Published:** 2025-09-03

**Authors:** Jiawei Huang, Bei Wang, Bomeng Wu

**Affiliations:** ^1^Department of Thoracic Surgery, Affiliated Gaozhou People’s Hospital, Guangdong Medical University, Maoming, Guangdong, China; ^2^Guangdong Medical University, Zhanjiang, Guangdong, China

**Keywords:** non-small cell lung cancer, anlotinib, adenocarcinoma, first-line treatment, low-dose therapy

## Abstract

This case report describes a 49-year-old male patient with advanced lung adenocarcinoma (cT3N3M1b, stage IVA), brain metastases, an ECOG PS of 3, negative driver gene status and low PD-L1 expression (TPS 1%). Traditional treatment options were limited. Following a multidisciplinary team consultation, the patient was prescribed a low dose of anlotinib (8 mg/day on d1–14, q21d) as a first-line treatment. Following two cycles of treatment, there was a significant improvement in symptoms (muscle strength recovery and the ability to walk independently), and an imaging assessment revealed shrinkage of the primary tumour and brain metastases. After seven cycles, the ECOG PS improved to 0, but after 11 cycles, progression occurred (tumour enlargement and new effusion). Switching to a combination of pemetrexed disodium and bevacizumab resulted in another response. The patient’s overall survival reached 16 months (anlotinib PFS: 9 months). This case suggests that low-dose anlotinib may serve as a bridging therapy for patients with advanced non-small cell lung cancer (NSCLC) and a poor general condition, improving their physical status and creating conditions for subsequent treatment. The potential of anlotinib for first-line application and strategies for overcoming drug resistance warrant further exploration.

## Introduction

Lung adenocarcinoma, the primary subtype of non-small cell lung cancer (NSCLC), poses significant challenges in the treatment of advanced stages, particularly for patients with a poor performance status (ECOG PS ≥ 3) and negative driver gene status. These patients often cannot tolerate standard therapy and derive limited clinical benefit. This study reports on a patient in this category who received anlotinib as a first-line therapy.

## Case report

**Figure 1:**
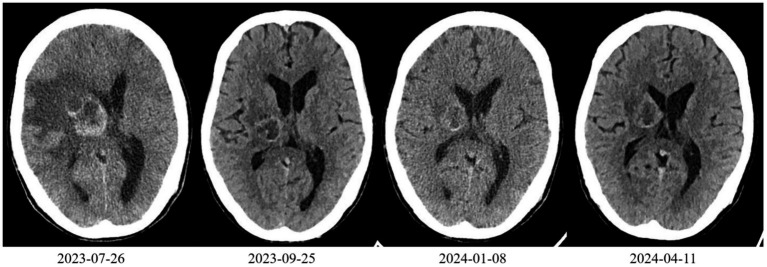
Cranial CT scan of the patient.

**Figure 2:**
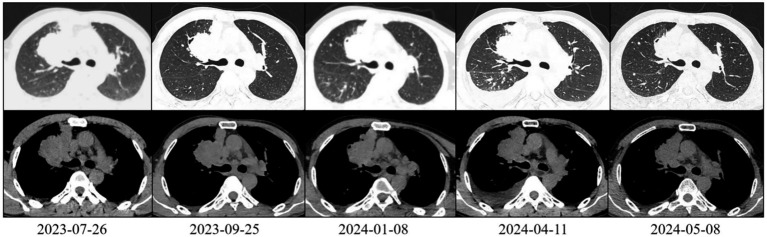
Chest CT scan of the patient (lung and mediastinal windows).

On 26 July 2023, a 46-year-old male presented to Gaozhou People’s Hospital with left-sided limb weakness that had persisted for over a month. His ECOG PS was 3 and he had bilateral supraclavicular lymph node enlargement and left-sided limb muscle strength grade I. He had no history of smoking, alcohol consumption or other relevant medical conditions. CT findings: A space-occupying lesion in the right cerebral hemisphere (5.0 cm × 3.1 cm) (Figure 1). There was also a space-occupying lesion in the right upper lung (6.8 cm × 5.2 cm) (Figure 2), with multiple enlarged lymph nodes in the mediastinum, bilateral hilar regions and bilateral supraclavicular fossae. No tumours were detected in the liver, adrenal glands or bones. Serological testing: Elevated levels of the following markers were observed: Carcinoembryonic antigen (CEA): 7.65 ng/mL (reference range: 0–5.20); Neuron-specific enolase (NSE): 24.80 ng/mL (reference range: 0–16.30); Cytokeratin 19 fragment antigen 21-1 (CYFRA21-1): 4.40 ng/mL (reference range: 0–3.30); Squamous cell carcinoma antigen (SCC): 1.31 ng/mL (reference range: 0–2.70). Bronchoscopy revealed a mass in the right upper lung. The pathological biopsy result was moderately differentiated invasive lung adenocarcinoma.

The patient was diagnosed with lung adenocarcinoma (cT3N3M1b, stage IVA, 8th AJCC). Next-generation sequencing revealed negative molecular genetic test results for EGFR, ALK, ROS1, BRAF, RET, MET, KRAS, NRAS and ERBB2. Only a TP53 p. E285* nonsense mutation was detected, and low PD-L1 expression (TPS 1%). Following a multidisciplinary team consultation and obtaining consent from the patient and his family, we decided to administer a low dose of anlotinib (8 mg, d1-14, q21d).

On 25 September 2023, after two cycles of treatment, the patient’s symptoms had improved significantly. His lower limb muscle strength had recovered, enabling him to walk independently instead of using a wheelchair. An imaging assessment revealed that the brain metastasis tumour measured 3.3 cm × 2.8 cm (Figure 1). The primary tumour measured 6.2 cm × 4.0 cm and showed new cavitary changes (Figure 2).

On 8 January 2024, after seven treatment cycles, the patient had improved muscle strength in the left upper limb and was able to walk independently, carry heavy objects and perform fine motor skills. The ECOG PS score was 0. Imaging assessment showed that the brain metastasis tumour measured 2.8 cm × 2.6 cm (Figure 1), while the primary tumour measured 5.4 cm × 3.9 cm (Figure 2).

On 11 April 2024, after 11 cycles of treatment: The patient’s symptoms remained unchanged with an ECOG PS score of 1. Serological testing/tumour markers: CEA: 11.20 ng/mL, NSE: 42.10 ng/mL, CYFRA21-1: 7.98 ng/mL. Imaging assessment: Brain metastasis measures 2.9 cm × 2.8 cm (Figure 1). The primary lung tumour measures 6.0 cm × 4.1 cm (Figure 2). There is a small amount of newly detected pleural effusion on the right side and moderate pericardial effusion. We concluded that the tumour had progressed and had become resistant to anlotinib. Following a consultation with the multidisciplinary team, we performed a pericardial puncture and drainage to relieve symptoms of fluid accumulation. The antitumour treatment regimen was then adjusted to include 800 mg of pemetrexed disodium and 600 mg of bevacizumab intravenously every 3 weeks.

On 6 May 2024, 20 days after chemotherapy, the tumour markers were as follows: CEA: 10.40 ng/mL, NSE: 35.10 ng/mL, CYFRA21-1: 2.98 ng/mL, SCC: 1.50 ng/mL. The imaging assessment revealed a primary lung tumour measuring 5.6 cm × 2.4 cm with no fluid accumulation (Figure 2), indicating treatment efficacy. Treatment with the original regimen (pemetrexed disodium + bevacizumab) continued on 9 May 2025.

Following two cycles of treatment with pemetrexed disodium + bevacizumab, the patient did not undergo any further treatment or examinations. Follow-up continued until the patient died in November 2024. The patient’s overall survival (OS) was 16 months, and progression-free survival (PFS) was 9 months during anlotinib treatment. The characteristics of patients at different stages were summarised in Table 1.

**Table 1:** Date and characteristics.

Characteristics	26 July 2023	25 September 2023	8 January 2024	11 April 2024	6 May 2024
Symptom	Left hemiplegia	Walking independently	Handling heavy objects, performing fine motor skills	Chest tightness, normal activity	normal activity
ECOG PS	3	1	0	1	1
Brain metastasis tumour	5.0 cm × 3.1 cm	3.3 cm × 2.8 cm	2.8 cm × 2.6 cm	2.9 cm × 2.8 cm	NM
Primary lung tumour	6.8 cm × 5.2 cm	6.2 cm × 4.0 cm	5.4 cm × 3.9 cm	6.0 cm × 4.1 cm	5.6 cm × 2.4 cm
Effusion	No	No	No	Pleural and pericardial effusion	Disappear
CEA (ng/mL)	7.65 (↑)	NM	NM	11.2 (↑)	10.4 (↑)
NSE (ng/mL)	24.80 (↑)	NM	NM	42.1 (↑)	35.1 (↑)
CYFRA21-1 (ng/mL)	4.40 (↑)	NM	NM	7.98 (↑)	2.98 (−)
SCC (ng/mL)	1.31 (−)	NM	NM	NM	1.50 (−)
Efficacy evaluation	/	PR	SD	PD	SD
Therapeutic regimen	/	After 2 cycles of anlotinib	After 7 cycles of anlotinib	After 11 cycles of anlotinib	20 days after treatment with pemetrexed disodium and bevacizumab

CEA, Carcinoembryonic antigen; NM, Not mentioned; NSE, neuron-specific enolase; PD, disease progression; PR, partial response; SCC, squamous cell carcinoma; SD, stable disease.

## Discussion

Lung cancer is the most common malignant tumour worldwide in terms of both incidence and mortality (1), with NSCLC accounting for 80–85% of cases (2, 3). Lung adenocarcinoma represents one of the major subtypes of NSCLC, and in the context of advanced lung adenocarcinoma, the identification of tumour driver genes and the assessment of patient performance status are pivotal in determining treatment decisions.

The patient in this case had been diagnosed with lung adenocarcinoma with brain metastasis and was negative for driver genes, with a generally poor condition. In accordance with conventional treatment methodologies, he would have been administered best supportive care. Following a comprehensive evaluation of the extant opinions on the subject, a novel approach was adopted, namely the implementation of low-dose anlotinib as a first-line treatment (8 mg/day, a reduction from the conventional 12 mg/day).

Anlotinib is an orally administered multi-targeted tyrosine kinase inhibitor (TKI) with anti-angiogenic and tumour proliferation-inhibiting effects. The drug has been approved in China for the treatment of advanced or metastatic NSCLC in patients who have previously received a minimum of two lines of chemotherapy. The ‘ALTER 0303’ study indicated that for patients with advanced NSCLC who progressed after second-line or further treatment, the OS was 9.6 months, the PFS was 5.4 months, the objective response rate (ORR) was 9.2%, and the disease control rate (DCR) was 81.0% (4). In this particular instance, the patient demonstrated an OS period of 16 months. It is hypothesised that the observed outcomes may be attributable to the patient receiving anlotinib as a primary treatment modality, subsequently followed by further anticancer interventions. Conversely, the phenomenon may also be ascribed to individual variability.

The patient underwent first-line treatment with anlotinib, after which his lung tumours and brain metastases gradually shrank, while his limb muscle strength significantly improved. Although anlotinib is reported to cause adverse reactions such as hypertension, hand-foot syndrome, thyroid dysfunction and so on (4, 5), none of these occurred in this case. Following several treatment cycles, the patient’s symptoms improved significantly and tumour shrinkage was observed, indicating a partial response (PR). However, follow-up examinations after 11 cycles indicated tumour growth, suggesting anlotinib resistance. Following re-evaluation and a multidisciplinary consultation, it was determined that the patient could tolerate chemotherapy. The treatment regimen was therefore changed to include pemetrexed disodium and bevacizumab. Follow-up examinations after the change in treatment showed that the tumour had shrunk further, indicating that alternative anticancer regimens remain effective after anlotinib treatment.

Anlotinib demonstrated a noteworthy bridging role in this particular instance, thereby substantiating its potential as a first-line treatment for NSCLC. In cases of patients exhibiting a suboptimal general condition, the administration of low-dose anlotinib therapy has been demonstrated to enhance their physical state, thereby establishing a conducive environment for subsequent therapeutic interventions. This case further demonstrates that alternative anticancer regimens may remain effective even after the development of anlotinib resistance. In the context of tumour treatment, this approach may constitute an innovative therapeutic strategy, offering a positive outlook for patients diagnosed with advanced NSCLC and enhancing their quality of life while living with the disease.

It is hypothesised that anlotinib, through its capacity to reduce tumour size and enhance physical condition, has the potential to serve as a transitional option for patients with NSCLC who are unable to tolerate standard treatment in the future. However, further case data and related studies are required to explore the potential of anlotinib as a first-line treatment for patients with poor general condition, as well as to investigate the efficacy and safety of low-dose regimens.

## Data Availability

The raw data supporting the conclusions of this article will be made available by the authors, without undue reservation.
